# Nursing Progress and Its Developments: VII. The Probationer's Third Year

**Published:** 1918-02-02

**Authors:** 


### THE MATRONS' AND SISTERS' DEPARTMENT.

### NURSING PROGRESS AND ITS DEVELOPMENTS.

### VII. The Probationer's Third Year.

THE third year is in many respects the most important of the whole course. It leads up to the final examination which decides the nurse's career, and regular time should therefore be spent in study during the whole twelve months to obviate cramming at the end. Moreover, the probationer is now in a position to benefit by advanced courses of instruction given by members of the honorary medical and surgical staff, such as she will never again have an opportunity of hearing. With all this she has become of great value to the institution. In most training-schools she enters upon the duties of staff-nurse at the commencement of this year, though not always dignified by this title. She takes charge of the ward in the absence of the sister, and helps in the teaching of probationers. In this year, too, she gets practical experience in the theatre, if she is lucky; and if not, in the out-patient or casualty departments. Twelve months are not too much for these varied experiences, and for the gradual growth of nursing responsibility which springs at this time.

The position of the London Hospital in restricting the formal training of its probationers to two years is now almost unique, and it demands some comment. The course allows eighteen months for the work usually spread over two years, and six months for the period sketched out above in which the probationer perfects herself in preparation for her final examination. The dangers of thus speeding up the training are obvious. But it is claimed at the London, first, that the unrivalled nursing opportunities afforded to probationers in this institution have the effect of making the shorter period of training equivalent to the longer one given in average establishments; secondly, that extreme concentration on the business of learning is made possible by the size and organisation of the trained staff in this training-school; and lastly, that the supervision exercised over the members of the private staff for the succeeding two years of the term during which all probationers are pledged to continue in the service of the hospital, and their frequent return to the wards, as an integral part of the system, constitute in effect a continuation of training. No one would desire to dispute the excellence of the nurses turned out under these conditions. But the drawback remains that the system, however admirable, has deprived the London Hospital of the honour of leading nursing opinion, since it demands for its success a type of institution and of organisation which is admittedly unique and incapable of being imitated.

The College of Nursing has taken the nursing standard where it found it, and no fairer procedure could have been adopted. Except for nurses whose training was completed before 1899 (for whom a lower standard has been agreed upon) the candidates for registration by the College fall under three headings. First there are nurses trained at certain recognised and *affiliated* hospitals and recognised Poor-Law infirmaries, and these must present the certificate of three years' training given in such institutions. Secondly, there are nurses trained in a recognised nurse-training school *or schools* who must hold a certificate *or certificates* of three years' training. Thirdly, there are nurses holding a certificate of not less than two years in a nurse-training school recognised by the Council, followed by at least two years of *bona-fide* practice as a nurse.

From these conditions it is apparent that the College is indisposed to accept broken training made up of a year or more passed at a hospital offering a three years' course, and pieced out at other institutions. If such terms of training were accepted, institutions would have no security that nurses would not drift about at will. Next it will be noted by many nurses with great satisfaction that women who hold certificates from hospitals offering only a two-year course, and who have supplemented their training by at least one year of regular training elsewhere, are eligible for registration. For the present, too, the College recognises the position of women who hold only the certificate of training-schools with two-year courses, but it enforces the rule that such certificates must be followed by two years' practice as a nurse before they can be considered valid.

For the time being all the complicated questions relating to training at special hospitals are left untouched. The greatest possible care has been observed not to injure the prospects of any woman whose training, though it may be considered to fall short of the highest standard attainable, has yet been carried out in conformity with the established curriculum in reputable training-schools.

